# Delayed spinal endoscopy-assisted posterolateral removal of deep retained glass fragments at the pediatric cervicothoracic junction after emergency debridement: a case report

**DOI:** 10.1186/s12893-026-04042-4

**Published:** 2026-08-04

**Authors:** Mingyang Wei, Jiantao Shi, Yi Dai, Lei Jiao, Xinyumeng Yang, Jianhua Sun

**Affiliations:** First Affiliated Hospital of Shihezi University, Shihezi, Xinjiang Uygur Autonomous Region China

**Keywords:** Pediatric trauma, Penetrating neck injury, Cervicothoracic junction, Glass foreign body, Delta spinal endoscopy, Case report

## Abstract

**Background:**

Deep retained glass foreign bodies at the pediatric cervicothoracic junction are rare and technically challenging. This region is close to the neural foramen, nerve roots, pleural apex, deep vessels, and osseous structures. During the contaminated emergency phase, aggressive exploration in pursuit of complete radiological clearance may increase the risk of iatrogenic injury. A staged strategy may therefore be considered when selected high-risk fragments cannot be safely removed during initial debridement.

**Case presentation:**

We report the case of a 13-year-old boy who sustained an open glass injury to the left side of the neck in a traffic accident. Initial computed tomography (CT) showed multiple hyperdense foreign bodies in the left posterolateral neck and cervicothoracic soft tissues. The deeper fragments were adjacent to the left second costotransverse region and in close proximity to the left T1–T2 intervertebral foramen. Cervical computed tomography angiography (CTA) and venography (CTV) identified no major vascular injury. Emergency debridement and exploration were performed through the original wounds, and several accessible large glass fragments were removed. Deep fragments that could not be safely accessed were not pursued by blind or extended exploration. Three months later, follow-up CT showed persistent, relatively large retained glass fragments adjacent to the left second costotransverse region. After detailed imaging assessment and preoperative localization, delayed removal was performed using a Delta spinal endoscopic system through a posterolateral working channel established via the left T1–T2 intertransverse space. Two large deep glass fragments and multiple irregular fragments were removed under magnified endoscopic visualization. Postoperative CT confirmed removal of the target deep foreign bodies, with only scattered tiny hyperdense foci remaining. The child recovered uneventfully, with good wound healing and normal sensation and muscle strength in both upper limbs.

**Conclusion:**

This case illustrates that, after emergency debridement, delayed Delta spinal endoscopy-assisted posterolateral removal may be considered for selected deeply retained glass fragments at the pediatric cervicothoracic junction when repeat imaging confirms stable localization and a safe working corridor. Small, scattered, asymptomatic residual fragments may be managed with close follow-up.

## Introduction

Penetrating neck injuries are relatively uncommon in clinical practice; however, their assessment and management are high-risk because of the narrow anatomical confines of the neck and its proximity to major neurovascular structures, the trachea, esophagus, and important osseous elements. In recent years, the management of penetrating neck trauma has gradually shifted from mandatory exploration based on traditional anatomical zones to a selective strategy guided by hemodynamic status, clinical findings, injury trajectory, and imaging results such as computed tomography angiography (CTA) [1, 2]. In children and adolescents, the soft-tissue layers of the neck are relatively thin and the anatomical spaces are smaller. When a foreign body extends deeply into the cervicothoracic junction, surgical exposure and safe removal become even more challenging.

Glass foreign body injuries differ from stab wounds or injuries caused by a single metallic foreign body. They are often characterized by multiple fragmented pieces, irregular morphology, sharp edges, and complex injury trajectories. Glass fragments are usually visible on plain radiographs or computed tomography (CT), while CT can further delineate the spatial relationship between the foreign body and surrounding vessels, nerves, bony structures, and the aerodigestive tract, thereby providing important information for surgical planning and risk assessment [3]. Previous reports have described penetrating cervical injuries caused by windshield glass fragments, as well as cases of glass foreign bodies impacted in the parapharyngeal space with neurovascular involvement. These reports highlight the need for thorough preoperative evaluation of the location of cervical glass foreign bodies and their relationship to adjacent structures, with individualized selection of surgical timing and approach based on risk assessment [4, 5].

At present, reports of deeply retained glass foreign bodies at the pediatric cervicothoracic junction remain limited, particularly when fragments are adjacent to the left second costotransverse region and left T1–T2 intervertebral foramen after emergency debridement. Reports of spinal endoscopic foreign body removal are also scarce and have mainly involved metallic foreign bodies, such as intraspinal bullets, shrapnel, or broken instruments, rather than pediatric glass fragments in the cervicothoracic soft-tissue corridor [6–8]. Here, we report a case of an open glass injury to the left neck in a child. We focus on the staged treatment strategy combining emergency debridement with delayed spinal endoscopic removal, and discuss the potential value and practical limitations of spinal endoscopy as a minimally invasive salvage option for deeply retained foreign bodies in the soft tissues of the cervicothoracic junction.

## Case presentation

**Fig. 1:**
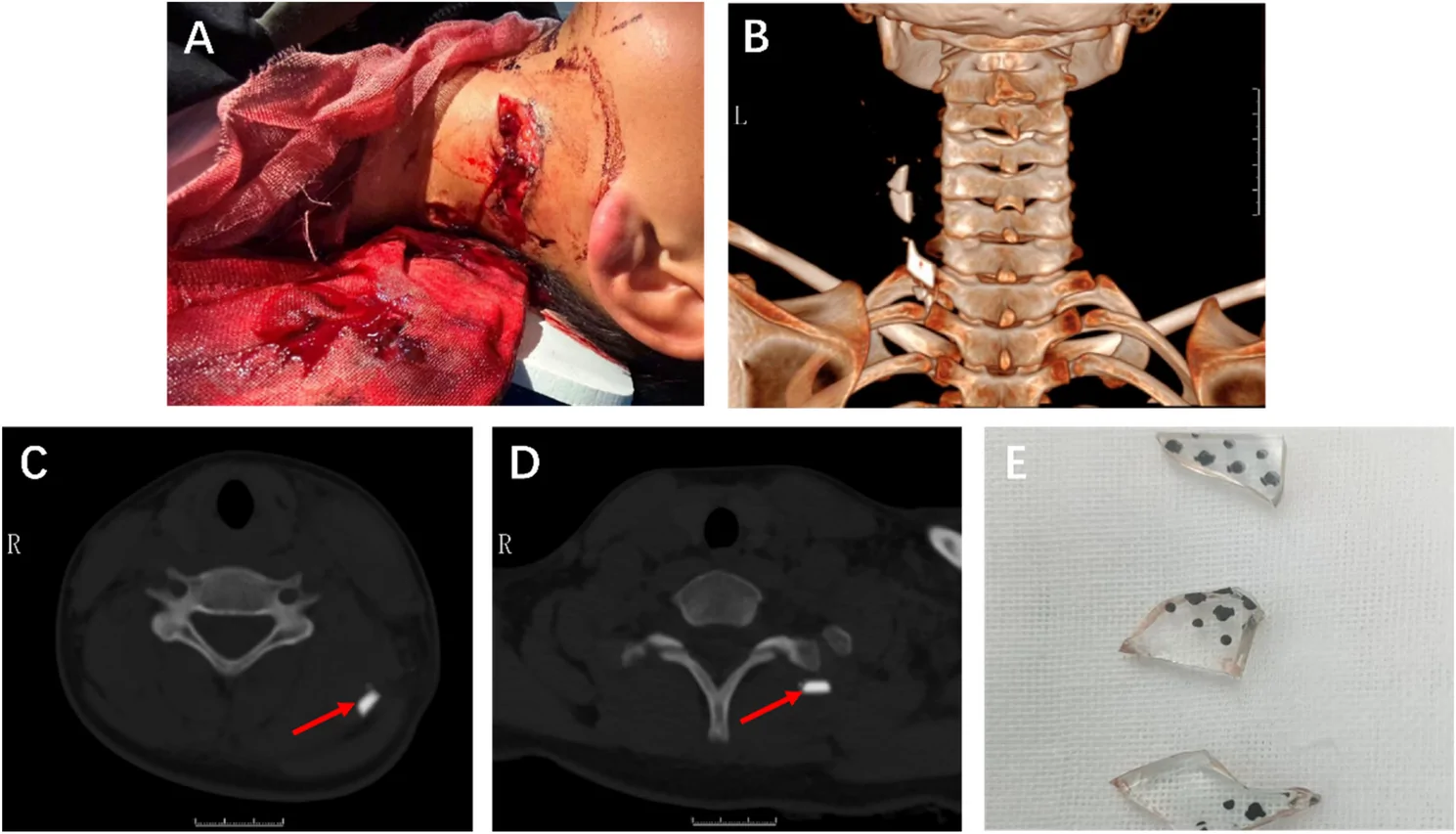
Initial presentation and emergency management.** A**: Open contaminated wounds on the left side of the neck at presentation. **B**: Preoperative volume-rendered CT reconstruction obtained during the emergency assessment, showing multiple hyperdense glass fragments extending from the left lower cervical region toward the left second costotransverse region. **C–D**: Axial CTA/CTV source images displayed using a bone window at different craniocaudal levels, showing glass fragments in the left posterolateral cervical and cervicothoracic soft tissues (red arrows). **E**: Representative glass fragments removed during first-stage wound debridement and exploration

A 13-year-old boy was admitted to the emergency department in October 2025 after a bicycle collision with a tricycle. During the accident, the left side of his neck struck a windshield, resulting in an open cervical wound with active bleeding. On admission, he was conscious and in a stable general condition, with stable vital signs. Neck movement was limited because of wound-related pain. Physical examination revealed two irregular open wounds on the left side of the neck, with ragged margins, obvious contamination, exposed subcutaneous soft tissue, and a small number of visible scattered foreign bodies locally (Fig. 1A). The wounds measured approximately 5 cm and 3 cm in maximal length, respectively. Muscle strength and tone in all four limbs were normal. No sensory deficit or pathological reflexes were detected.

Cervical CT demonstrated multiple hyperdense foreign bodies in the left posterolateral neck. Several deep fragments were located in the left posterolateral cervicothoracic soft tissues, adjacent to the left second costotransverse region and close to the left T1–T2 intervertebral foramen (Fig. 1B-D). No fragment was seen within the spinal canal, and no pneumothorax or pleural cavity penetration was identified on the available imaging. Further evaluation with cervical CTA and CTV identified no major arterial or venous injury. Because the fragments were irregular and partially overlapping on the available images, a reliable total fragment count and calibrated measurements of individual fragments could not be reconstructed retrospectively. Based on the history, physical examination, and imaging findings, the patient was diagnosed with an open injury of the left neck complicated by multiple retained glass foreign bodies.

The initial assessment focused on identifying potential injuries to major neurovascular structures and the aerodigestive tract, mapping the distribution of the glass fragments, identifying the deeply located fragments of greatest clinical concern, and clarifying their relationship with the left T1–T2 intervertebral foramen, transverse process, pleural apex, and the left second costotransverse region. The potential benefits and risks of deeper exploration during the emergency phase were also carefully considered. As CTA and CTV did not suggest major vascular injury and limb sensation and strength were intact, there was no evidence of overt vascular, spinal cord, or nerve root dysfunction. However, imaging showed multiple scattered glass fragments, some deeply located near bony structures of the cervicothoracic junction. Therefore, the goal of the initial emergency procedure was defined as bleeding control, thorough debridement, removal of visible and readily accessible major glass fragments, contamination control, and avoidance of blind exploration near the left T1–T2 intervertebral foramen, pleural apex, and deep vessels.

**Fig. 2:**
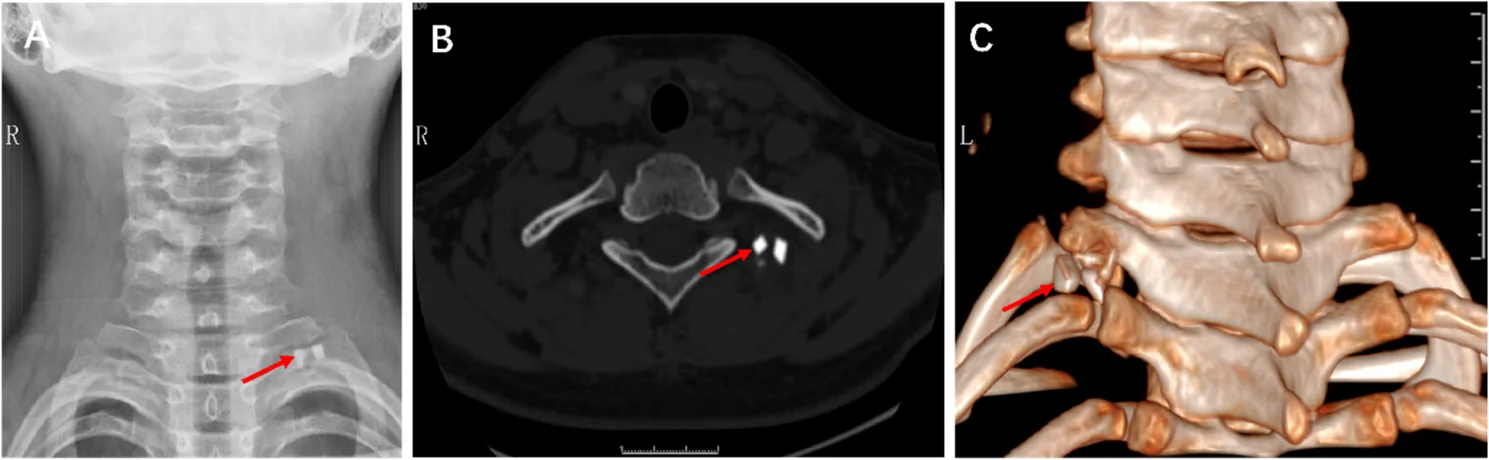
Retained glass fragments after first-stage debridement.** A**: Postoperative anteroposterior radiograph showing residual radiopaque fragments in the left lower cervicothoracic region (red arrow). **B**: Axial bone-window CT image after first-stage debridement showing retained glass fragments adjacent to the left second costotransverse region and in the vicinity of the left T1–T2 intervertebral foramen (red arrows). **C**: Postoperative three-dimensional CT reconstruction after the first-stage procedure showing the spatial relationship between the residual foreign bodies and the osseous structures of the cervicothoracic junction (red arrow)

Emergency wound debridement, exploration, and foreign body removal were performed. Through the two original wounds, three relatively large and potentially hazardous glass fragments were removed from the wound tract (Fig. 1E). The accessible fragments were located along the contaminated wound tract and were either visible or palpable during exploration. Contaminated and devitalized tissues were excised, followed by repeated irrigation and meticulous hemostasis. According to the preoperative CT findings, additional deeper glass fragments remained in the left posterolateral cervicothoracic soft tissues adjacent to the left second costotransverse region and near the left T1–T2 intervertebral foramen. Because the operative space in the deep portion of the original wound tract was limited and local soft-tissue oozing was evident, further incision extension and blind exploration were considered likely to increase the risk of injury to the nerve root, pleural apex, and deep vessels. There was also concern that additional manipulation could displace the glass fragments deeper. Therefore, forced retrieval was not attempted. After adequate hemostasis, the wound was closed in layers, and drainage was placed. Postoperatively, the patient remained hemodynamically stable. Sensation and muscle strength in all four limbs were normal, and no new neurological deficit occurred. He received antibiotic therapy, analgesia, wound care, drainage management, and other supportive therapy. Postoperative cervical radiography and CT showed no obvious abnormality of the cervical osseous structures, but scattered residual glass foreign bodies and a local hematoma remained in the left posterolateral cervical soft tissues and the space adjacent to the left second costotransverse region (Fig. 2). The patient was discharged after satisfactory wound recovery.

Three months after the initial procedure, the patient was readmitted because of persistent deep foreign bodies near the left second costotransverse region. Physical examination showed postoperative scarring on the left side of the neck without obvious local tenderness. Cervical and thoracic movements were not significantly restricted, and limb strength and sensation remained normal. Repeat cervical CT demonstrated persistent focal hyperdense foreign bodies in the left posterolateral cervicothoracic soft tissues and the space adjacent to the left second costotransverse region, without significant interval change. Although the patient had no evident infection, worsening pain, or neurological dysfunction at that time, the target foreign bodies were relatively large, sharp-edged, clearly localized, and close to the left T1–T2 intervertebral foramen and left second costotransverse region. Long-term retention was considered to carry potential risks, including mechanical irritation, migration, infection, and delayed neurovascular injury. The timing of the second-stage procedure was determined through a combination of clinical reassessment and shared decision-making with the patient’s guardians. By 3 months after the initial operation, the wound had healed, contamination and acute soft-tissue inflammation had subsided, repeat CT confirmed stable localization of the retained fragments, and a safer posterolateral working corridor could be planned. After the potential benefits and risks of continued observation versus surgical removal had been fully explained, the patient’s guardians preferred elective removal of the larger retained fragments and provided consent for a second-stage procedure. Therefore, the surgical team performed elective Delta spinal endoscopy-assisted posterolateral removal of the target deep glass fragments.

**Fig. 3:**
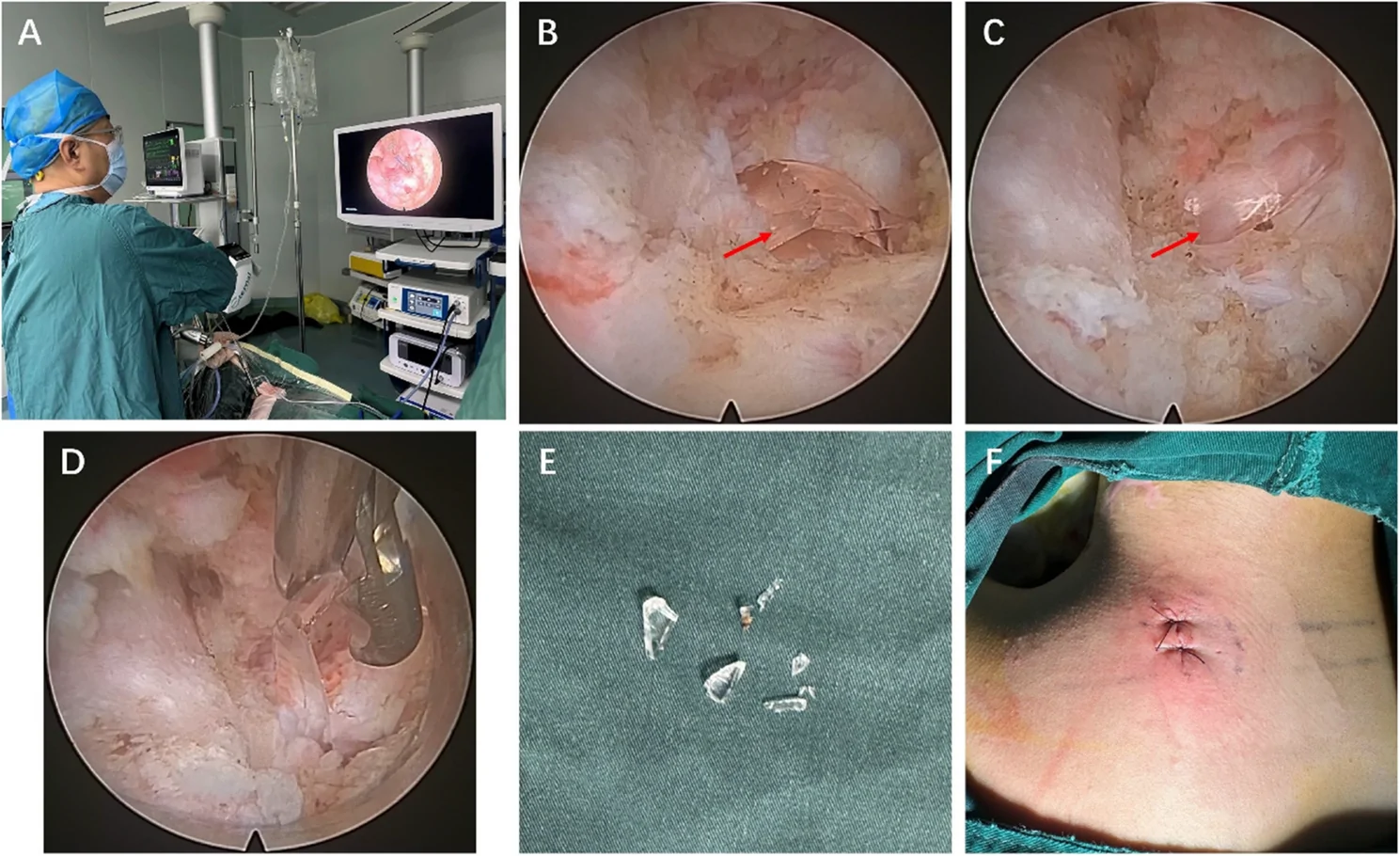
Second-stage spinal endoscopy-assisted removal of deeply retained glass fragments.** A**: With the patient in the prone position under general anesthesia, a Delta spinal endoscopic working channel was established through the left T1–T2 intertransverse space, followed by deep exploration under direct endoscopic visualization. **B–C**: Endoscopic views showing irregular glass fragments embedded within the deep soft-tissue planes (red arrows). **D**: Under magnified endoscopic visualization, the glass fragment was bluntly dissected, gradually mobilized, grasped with pituitary forceps, and removed intact. **E**: Multiple irregular glass fragments removed during the procedure. **F**: Postoperative posterolateral incision, illustrating the posterolateral minimally invasive approach

The second-stage operation was performed under general anesthesia with the patient in the prone position. Under fluoroscopic guidance, the left T1–T2 intertransverse space and the left second costotransverse region were localized. Anteroposterior fluoroscopy was used to identify the cervicothoracic junction and confirm the T1–T2 level, and a lateral image was used to confirm the planned working trajectory before final placement of the working channel. A longitudinal incision was made over the preoperatively marked left posterolateral target area and a Delta spinal endoscopic working cannula with an outer diameter of approximately 10 mm was introduced gradually. Under continuous endoscopic irrigation and magnified direct visualization, the soft tissues around the transverse process were dissected along the intermuscular planes. When necessary, a small amount of bone at the peripheral margin of the transverse process was carefully removed to improve exposure. Several irregular glass fragments were identified and removed sequentially. Exploration was then continued along the deep intermuscular plane toward the preplanned target region, where a relatively large glass fragment was found embedded within the muscular interval. After careful separation and mobilization with a nerve hook, the fragment was grasped with pituitary forceps and removed intact under magnified endoscopic visualization. Another large glass foreign body was subsequently removed from the lateral region in the same manner. The two larger glass fragments removed during the second-stage procedure were not measured intraoperatively with a sterile ruler. Based mainly on the calibrated CT images, together with postoperative gross appearance, the approximate dimensions of the two larger fragments were estimated to be 8 × 4 mm and 6 × 3.5 mm, respectively. The operative time was 55 min, and the estimated blood loss was 10 mL. Intraoperative neurophysiological monitoring was not used. Fluoroscopy was used only for anatomical localization at T1–T2 and working-channel adjustment. Although precise radiation-dose records were unavailable, the operative team estimated that fluoroscopy was limited to approximately three single-shot acquisitions: anteroposterior and lateral views for initial T1–T2 localization and one additional image during final working-channel placement. Cumulative fluoroscopy time and patient-specific radiation-dose data were not recorded and could not be calculated retrospectively. After confirming the absence of active bleeding, the operative field was thoroughly irrigated. The incision was closed in layers and dressed (Fig. 3).

**Fig. 4:**
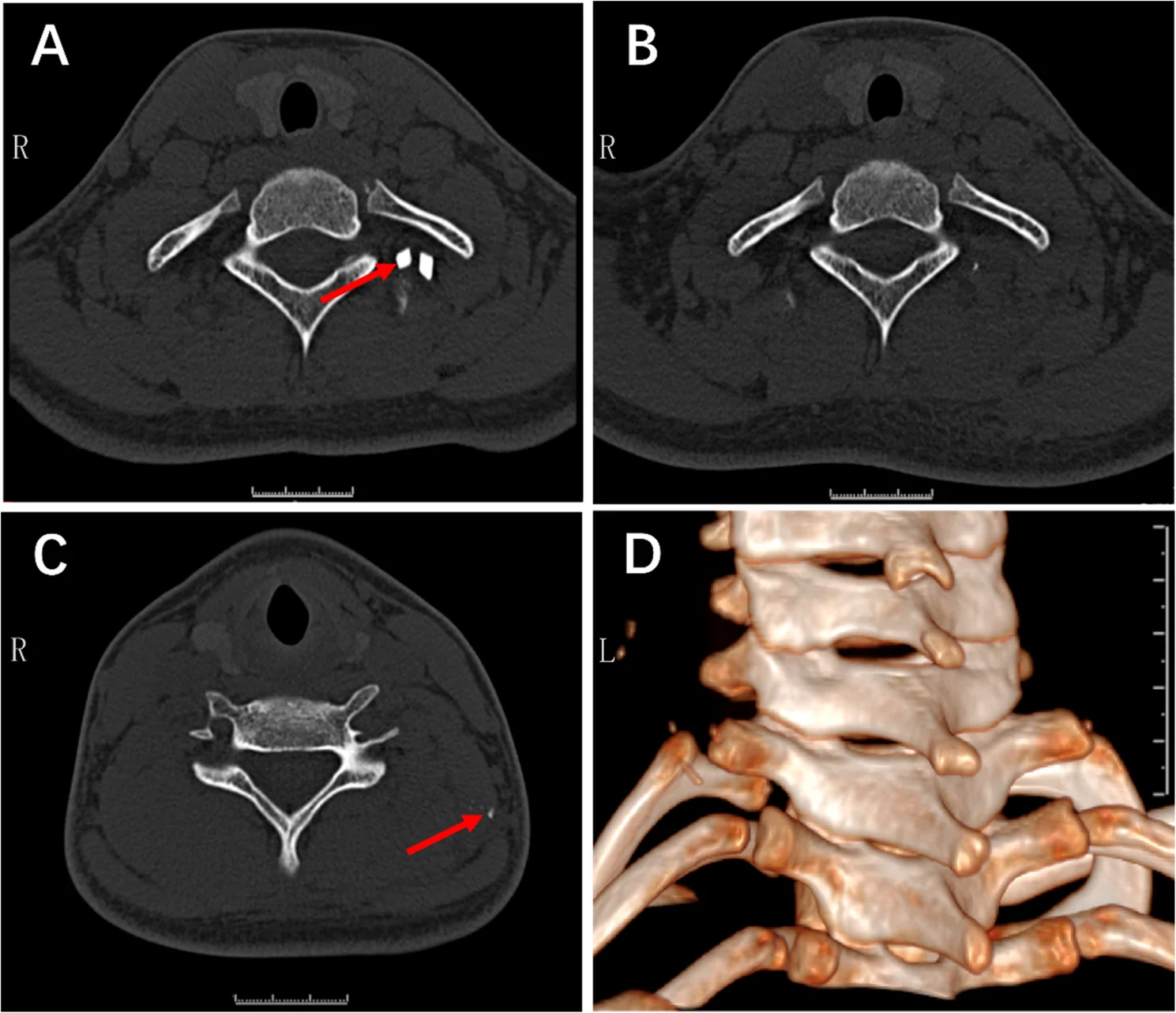
Imaging comparison before and after the second-stage procedure.** A**: Axial bone-window CT image before the second-stage procedure showing selected deep target glass fragments adjacent to the left second costotransverse region (red arrow). **B**: Postoperative axial bone-window CT image at the corresponding anatomic level showing no visible target fragment in the treated region. **C**: Postoperative axial CT image showing scattered tiny hyperdense foci in the left posterolateral cervicothoracic soft tissues (red arrow), considered to represent small residual glass fragments. **D**: Postoperative three-dimensional CT reconstruction after the second-stage procedure showing clearance of the selected target fragments from the left second costotransverse region

The patient recovered uneventfully after the second-stage procedure. Skin sensation and muscle strength in both upper limbs remained normal, with no numbness, weakness, or other new neurological symptoms. Postoperative CT showed that the larger deep foreign bodies adjacent to the left second costotransverse region had been removed, with a small amount of local postoperative fluid collection. Scattered tiny hyperdense foci remained in the left posterolateral cervicothoracic soft tissues, consistent with small residual glass fragments (Fig. 4). Given their small size, scattered distribution, and the absence of clinical symptoms, further extensive exploration was considered more likely to increase the risk of soft-tissue and neurovascular injury. After full discussion with the patient’s guardians, follow-up observation was chosen for the remaining tiny fragments. On postoperative day 3, the patient was in good general condition. The incision showed no redness, swelling, or exudation, and motor and sensory function in both upper limbs was normal. At the 3-month follow-up after the second-stage operation, the wound had healed well. The patient reported no neck or shoulder pain, numbness, weakness, or limitation in daily activities. Follow-up radiography showed no obvious change in the residual tiny hyperdense foci.

## Discussion

Penetrating neck injury with retained foreign bodies is uncommon in children but may lead to severe airway, vascular, neurological, infectious, or delayed mechanical complications. The pediatric neck and cervicothoracic junction contain compact and complex anatomical structures, and retained foreign bodies may lie close to major neurovascular structures, the pleural apex, the aerodigestive tract, neural foramina, and bony landmarks, making both diagnosis and treatment challenging. Previous reports of cervicothoracic penetrating foreign body injuries have emphasized the importance of detailed imaging assessment and individualized surgical planning [9]. In pediatric patients, the relatively narrow cervical anatomical space and dense distribution of vessels, airway structures, and nerves further increase the risk of severe complications after penetrating trauma [10, 11]. Published reports on deeply retained glass foreign bodies at the pediatric cervicothoracic junction remain scarce. In the available literature, cervical glass foreign bodies have more commonly been managed through conventional open approaches, whereas endoscopic removal of spinal or paraspinal foreign bodies has mainly been described for metallic objects, such as bullets, shrapnel, or broken surgical instruments, and mostly in thoracolumbar or lumbar regions [4–9]. The present case is therefore noteworthy because it describes the delayed, selective removal of deeply retained glass fragments in a child using a Delta spinal endoscopy-assisted posterolateral soft-tissue corridor adjacent to the left T1–T2 intervertebral foramen and second costotransverse region. Rather than proposing a broadly applicable technique, this case illustrates a potential minimally invasive salvage strategy.

The initial management in this case reflected a selective treatment strategy rather than an attempt to achieve complete radiological clearance during the acute contaminated phase. In the emergency setting, the primary objectives were hemostasis, wound debridement, contamination control, removal of accessible major foreign bodies, and protection of neurovascular structures. Glass foreign bodies are usually visible on radiographs or CT; however, when fragments are multiple, small, and scattered, some may become embedded in deep soft tissues, near osseous structures, or adjacent to important neurovascular structures, thereby increasing the risk of blind exploration. Adams et al. [2] emphasized that CT evaluation of penetrating neck injuries should follow a systematic approach, including assessment of the injury trajectory, vascular integrity, airway and digestive tract involvement, osseous injury, and associated complications. Voss et al. [3] also indicated that CT is useful for determining the material, location, and spatial relationship of foreign bodies to adjacent structures, thereby supporting surgical planning. In the present case, several large and accessible glass fragments were removed during the initial operation. For the deep fragments adjacent to the second costotransverse region and close to the left T1–T2 intervertebral foramen, further exploration during the acute contaminated phase could have increased the risk of nerve root injury, vascular injury, pleural violation, and excessive soft-tissue dissection. Therefore, deferring removal of selected deeply retained fragments was not incomplete treatment, but a deliberate risk–benefit decision.

The indication and timing of the second-stage procedure were individualized. Although the patient had no evident infection, progressive pain, or neurological dysfunction at the time of reassessment, the target fragments were relatively large, sharp-edged, clearly localized, and close to the left T1–T2 intervertebral foramen and costotransverse region. Long-term retention was considered to carry potential risks, including chronic mechanical irritation, foreign body reaction, migration, infection, and delayed neurovascular injury. Luo et al. [12] reported delayed rupture of the common carotid artery and internal jugular vein caused by a retained cervical foreign body after trauma. Yoshioka et al. [13] described migration of a glass fragment into the lumbar spinal canal, resulting in delayed pain and radicular symptoms. Ozsarac et al. [14] also reported clinically significant morbidity caused by delayed migration of a retained glass foreign body in soft tissue. These reports suggest that deeply located sharp foreign bodies may still pose a risk of delayed complications, even when early symptoms are minimal. Conversely, repeat surgery should also avoid overtreatment. For very small, scattered, deeply embedded, and asymptomatic residual fragments, forced removal may cause soft-tissue injury, bleeding, infection, or neurovascular damage that outweighs the expected benefit [15]. In the present case, the second-stage procedure was not intended to remove all residual fragments, but specifically targeted the relatively large, deeply located, and clearly localized foreign bodies with potential risk.

Several treatment alternatives were considered before selecting Delta spinal endoscopy-assisted posterolateral removal. Open re-exploration could have provided a wider operative field, but scar adhesion, the deep location of the fragments, proximity to the left T1–T2 intervertebral foramen and pleural apex, and the need for wider soft-tissue dissection could have increased surgical morbidity. CT-guided or fluoroscopy-guided percutaneous extraction may be useful for selected radiopaque foreign bodies, but grasping sharp glass fragments without direct visualization near foraminal and pleural structures may be hazardous. Ultrasound has an established role in detecting and localizing soft-tissue foreign bodies, including glass, and can also guide percutaneous removal in appropriate superficial or accessible cases [16, 17]. However, in this case, the target fragments were deeply located adjacent to osseous structures of the cervicothoracic junction, where acoustic shadowing from bone and proximity to the neural foramen may have limited the reliability and safety of ultrasound-guided extraction. The rationale for using Delta spinal endoscopy was therefore based on three considerations: the target fragments were well localized; a posterolateral intermuscular corridor through the left T1–T2 intertransverse space toward the left second costotransverse region could be planned; and endoscopy provided illumination, continuous irrigation, magnified visualization, and controlled instrument manipulation through a limited approach.

Published experience with endoscopic removal of spinal or paraspinal foreign bodies remains limited. Kravtsov et al. [6] reported full-endoscopic management of thoracolumbar bullet wounds, Karaeminogullari et al. [7] reported percutaneous transforaminal endoscopic removal of L5 foraminal shrapnel, and Guan et al. [8] described endoscopic retrieval of broken guidewires during percutaneous endoscopic lumbar surgery. Although these reports involved metallic foreign bodies and different anatomical regions, they support the technical concept that endoscopy can provide targeted direct-vision removal of selected deep spinal or paraspinal foreign bodies. The present case extends this concept to delayed removal of glass fragments through a pediatric cervicothoracic posterolateral soft-tissue corridor. However, this approach should not be generalized to all cervical or cervicothoracic foreign body injuries. Its use requires strict patient selection, detailed preoperative imaging, a clearly defined safe working corridor, and surgeons familiar with both spinal endoscopy and regional cervicothoracic anatomy.

Two technical safety issues deserve special consideration. First, fluoroscopy was used for anatomical localization at the T1–T2 level and working-channel adjustment. In this case, fluoroscopy was limited to a small number of single-shot acquisitions for T1–T2 localization and final working-channel placement. Minimally invasive spine procedures may require fluoroscopic guidance, and radiation exposure should be minimized by careful preoperative planning, intermittent rather than continuous fluoroscopy, collimation, protective shielding, and avoidance of unnecessary repeated imaging [18, 19]. This consideration is particularly important in pediatric patients. Second, the operation was performed near the left T1–T2 intervertebral foramen. Intraoperative neurophysiological monitoring can provide real-time information regarding motor and sensory pathway integrity during spine and spinal cord surgery, and motor evoked potentials and somatosensory evoked potentials are commonly considered when manipulation near neural structures carries substantial risk [20]. In the present case, intraoperative neurophysiological monitoring was not used because the procedure was performed outside the spinal canal through a posterolateral soft-tissue corridor, the patient had no preoperative neurological deficit, and the planned manipulation avoided direct neural decompression. Nevertheless, the absence of neuromonitoring should be acknowledged as a technical limitation, and neuromonitoring may be considered in future cases when the target foreign body is closer to the spinal canal, nerve root, or major neural elements.

This case has several limitations. First, as a single case report, it cannot establish the superiority of delayed Delta spinal endoscopy-assisted posterolateral removal over open surgery, image-guided percutaneous extraction, ultrasound-guided extraction, or continued observation. Second, only 3 months of follow-up after the second-stage procedure were available at the time of manuscript preparation; continued observation is required to determine whether the remaining tiny glass fragments may cause long-term foreign-body reaction, migration, infection, or delayed neurovascular irritation. Third, although the approximate dimensions of the two larger fragments removed during the second-stage procedure were estimated from calibrated CT images and postoperative gross appearance, intraoperative ruler-based measurement was not performed. The total initial fragment count, calibrated dimensions of all individual fragments, and a precise volumetric or three-dimensional quantitative comparison could not be reconstructed retrospectively because the fragments were irregular, partially overlapping, and not individually documented on the available images and operative record. Fourth, although the approximate fluoroscopy exposure pattern could be described, patient-specific fluoroscopy time and radiation-dose metrics were not retained and could not be calculated retrospectively. Accordingly, the findings of this report should be interpreted as an individualized treatment experience under specific anatomical and clinical conditions, rather than as a broadly applicable strategy for all retained glass foreign bodies in the neck or cervicothoracic junction.

## Conclusion

This case illustrates a staged, selective strategy for pediatric cervicothoracic glass foreign bodies. During the acute contaminated phase, treatment focused on hemostasis, debridement, contamination control, and safe removal of accessible fragments rather than complete radiological clearance. After wound healing and repeat imaging confirmed stable localization, delayed Delta spinal endoscopy-assisted posterolateral removal enabled targeted extraction of selected deep fragments adjacent to the T1–T2 intervertebral foramen and second costotransverse region. This approach may be considered only in carefully selected cases with a clearly defined safe working corridor and appropriate endoscopic expertise; small, scattered, asymptomatic residual fragments may be observed with follow-up.

## Data Availability

No datasets were generated or analysed during the current study.

## Abbreviations

CT: Computed tomography
CTA: Computed tomography angiography
CTV: Computed tomography venography
